# Overexpression of *ca1pase* Decreases Rubisco Abundance and Grain Yield in Wheat^1^

**DOI:** 10.1104/pp.19.00693

**Published:** 2019-07-31

**Authors:** Ana Karla M. Lobo, Douglas J. Orr, Marta Oñate Gutierrez, P. John Andralojc, Caroline Sparks, Martin A.J. Parry, Elizabete Carmo-Silva

**Affiliations:** a Lancaster University, Lancaster Environment Centre, Lancaster, LA1 4YQ, United Kingdom; b Federal University of Ceará, Department of Biochemistry and Molecular Biology, Fortaleza, Brazil; c Rothamsted Research, Plant Sciences Department, Harpenden, AL5 2JQ, United Kingdom

## Abstract

Rubisco catalyzes the fixation of CO_2_ into organic compounds that are used for plant growth and the production of agricultural products, and specific sugar-phosphate derivatives bind tightly to the active sites of Rubisco, locking the enzyme in a catalytically inactive conformation. 2-carboxy-d-arabinitol-1-phosphate phosphatase (CA1Pase) dephosphorylates such tight-binding inhibitors, contributing to the maintenance of Rubisco activity. Here, we investigated the hypothesis that overexpressing *ca1pase* would decrease the abundance of Rubisco inhibitors, thereby increasing the activity of Rubisco and enhancing photosynthetic performance and productivity in wheat (*Triticum aestivum*). Plants of four independent wheat transgenic lines overexpressing *ca1pase* showed up to 30-fold increases in *ca1pase* expression compared to the wild type. Plants overexpressing *ca1pase* had lower numbers of Rubisco tight-binding inhibitors and higher Rubisco activation state than the wild type; however, there were 17% to 60% fewer Rubisco active sites in the four transgenic lines than in the wild type. The lower Rubisco content in plants overexpressing *ca1pase* resulted in lower initial and total carboxylating activities measured in flag leaves at the end of the vegetative stage and lower aboveground biomass and grain yield measured in fully mature plants. Hence, contrary to what would be expected, *ca1pase* overexpression decreased Rubisco content and compromised wheat grain yields. These results support a possible role for Rubisco inhibitors in protecting the enzyme and maintaining an adequate number of Rubisco active sites to support carboxylation rates in planta.

## Abstract (precis)

ca1pase overexpression decreased the content of Rubisco inhibitors and the number of Rubisco active sites in wheat leaves, with consequent decreases in biomass and grain yield.

Rates of yield increase for major food crops have recently slowed and in some cases stagnated, spurring efforts to identify approaches to reverse this trend (Long et al., 2015). Despite the benefits brought about by breeding programs, together with better farming practices implemented in the last century, current predictions suggest that an increase in agricultural production of 70% will be required to support the projected demand over the coming decades (Tilman et al., 2011; Ray et al., 2013). Global food security will also be increasingly challenged by fluctuations in crop production resulting from climate change (Ray et al., 2015; Tilman and Clark, 2015), for example, through altered soil-atmosphere and plant-atmosphere interactions (Dhankher and Foyer, 2018). The development of high-yielding and climate-resilient food crops is thus emerging as one of the greatest global challenges to humankind (Long et al., 2015; Paul et al., 2017).

Plant growth and biomass production are determined by photosynthetic CO_2_ assimilation, a process with scope for significant improvement (Zhu et al., 2010). In recent years, improving photosynthesis has emerged as a promising strategy to increase crop yields without enlarging the area of cultivated land (Ort et al., 2015). A number of recent studies have been successful in the use of genetic manipulation of photosynthetic enzymes to improve genetic yield potential by increasing carbon assimilation and biomass production (Nuccio et al., 2015; Simkin et al., 2015; Kromdijk et al., 2016; Driever et al., 2017).

Ribulose-1,5-bisphosphate (RuBP) carboxylase/oxygenase (Rubisco) catalyzes the first step in the Calvin-Benson-Bassham cycle, fixing CO_2_ through the carboxylation of RuBP. Modulation of Rubisco activity is complex and involves interaction with many cellular components (see reviews by Andersson, 2008; Parry et al., 2008). We have postulated that regulation of the carboxylating enzyme in response to the surrounding environment is not optimal for crop production (Carmo-Silva et al., 2015). Estimates from modeling and in vivo experimentation suggest that improving the regulation of Rubisco activity has the potential to improve carbon assimilation by as much as 21% (Reynolds et al., 2009; Taylor and Long, 2017).

Certain phosphorylated compounds bind tightly to Rubisco active sites, locking the enzyme in a catalytically inactive conformation (see Bracher et al., 2017). These inhibitors include 2-carboxy-d-arabinitol-1-phosphate (CA1P), a naturally occurring Rubisco inhibitor that is produced in the leaves of some plant species under low light or darkness (Gutteridge et al., 1986; Moore and Seemann, 1992). In addition, catalytic misfire (i.e. the low-frequency but inexorable occurrence of side reactions within the catalytic site of Rubisco, described by Pearce, 2006) occurs during the multistep carboxylase and oxygenase reactions catalyzed by Rubisco. These side reactions lead to production of phosphorylated compounds that resemble the substrate RuBP and/or reaction intermediates. Misfire products, including xylulose-1,5-bisphosphate (XuBP) and d-glycero-2,3-pentodiulose-1,5-bisphosphate, bind tightly to either carbamylated or uncarbamylated active sites, inhibiting Rubisco activity (Parry et al., 2008; Bracher et al., 2017).

Inhibitor-bound Rubisco active sites are reactivated by the combined activities of Rubisco activase (Rca) and specific phosphatases, such as CA1P phosphatase (CA1Pase) and XuBP phosphatase, in a light-dependent manner. Rca remodels the conformation of active sites to facilitate the release of inhibitors; CA1Pase and XuBP phosphatase convert the sugar-phosphate derivatives into noninhibitory compounds by removing the phosphate group (Andralojc et al., 2012; Bracher et al., 2015).

Of all the naturally occurring Rubisco inhibitors, CA1P is the only one known to be actively synthesized, while the others are byproducts of Rubisco activity. The light/dark regulation of Rubisco activity by CA1P has received considerable attention in a number of studies since the nocturnal inhibitor was first described (Gutteridge et al., 1986; Berry et al., 1987; Holbrook et al., 1992; Moore and Seemann, 1994). Nonaqueous subcellular fractionation (Parry et al., 1999) and metabolic studies (Andralojc et al., 1994, 1996, 2002) have shown that CA1P is produced in the chloroplast by phosphorylation of 2-carboxy-d-arabinitol (CA) during low light or darkness, while CA is derived from light-dependent reactions: CO_2_ → Calvin cycle → chloroplastic Fru bisphosphate → hamamelose bisphosphate → 2Pi + hamamelose/2-hydroxymethylribose → CA. CA1P binds tightly to carbamylated Rubisco active sites (Moore and Seemann, 1994). In an ensuing period of illumination, CA1P is released from Rubisco by the action of Rca and is then dephosphorylated by CA1Pase in a pH- and redox-regulated process (Salvucci and Holbrook, 1989; Andralojc et al., 2012) to yield the noninhibitory products CA and Pi.

Some plant species contain only modest amounts of CA1P. For example, Moore et al. (1991) showed that dark-adapted leaves of wheat (*Triticum aestivum*) contain sufficient CA1P to inhibit no more than 7% of the available Rubisco active sites. By contrast, comparable leaves of species from the genera *Petunia* and *Phaseolus* contain sufficient CA1P to occupy all available Rubisco catalytic sites (Moore et al., 1991). Even so, both wheat and *Phaseolus vulgaris* (and all other land plant species so far investigated) possess the gene for CA1Pase (Andralojc et al., 2012). The presence of the capacity to synthesize and remove CA1P, even in species which do not produce sufficient CA1P to significantly influence whole leaf Rubisco activity, implies that CA1P may be more than a simple regulator of Rubisco activity.

Daytime inhibitors of Rubisco activity present in wheat leaves have proven too unstable for detailed study (Keys et al., 1995). However, Andralojc et al. (2012) showed that CA1Pase efficiently dephosphorylates sugar-phosphate derivatives closely related to CA1P, such as CA 1,5-bisphosphate and 2-carboxy-d-ribitol 1,5-bisphosphate (CRBP), and that CA1Pase also appears to dephosphorylate the main contender for diurnal inhibition of Rubisco, d-glycero-2,3-pentodiulose-1,5-bisphosphate (Kane et al., 1998).

In vitro experiments provide evidence that CA1P may protect Rubisco from proteolytic breakdown under stress conditions (Khan et al., 1999), in addition to any role it may play as a reversible regulator of Rubisco catalytic activity. However, the in vivo significance of this potential protective role is unknown. Most published studies have focused on the in vitro regulation of Rubisco activity by inhibitors and CA1Pase (Berry et al., 1987; Parry et al., 1997; Kane et al., 1998; Khan et al., 1999; Andralojc et al., 2012). Charlet et al. (1997) showed that CA1Pase abundance is species specific but generally represents <0.06% of the leaf total protein concentration.

In the current study, we investigated the hypothesis that overexpression of *ca1pase* would lower the content of Rubisco inhibitors and, consequently, increase Rubisco activation state, Rubisco activity, CO_2_ assimilation, and grain yield production. We demonstrate that *ca1pase* overexpression does decrease the quantity of Rubisco inhibitors in vivo, but it also decreases the number of Rubisco active sites in wheat leaves and reduces biomass production and grain yield. These results imply that the multiple elements involved in the regulation of Rubisco activity must be carefully balanced during attempts to improve crop productivity by genetically engineering this complex photosynthetic enzyme.

## RESULTS

### Transgenic Wheat Lines Overexpressing *ca1pase*

**Figure 1.**
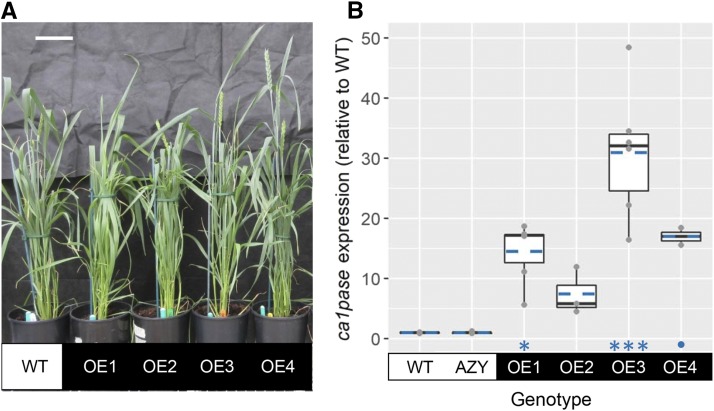
Wheat transgenic lines overexpressing *ca1pase*. A, Plants grown under well-watered conditions in a glasshouse. Measurements and pictures were taken before anthesis. Scale bar = 10 cm. B, Relative expression of *ca1pase* in wild-type plants (WT), negative controls (AZY), and transgenic lines overexpressing *ca1pase* (OE1–OE4). Boxes represent the median and the first and third quartiles, and whiskers represent the range; symbols represent individual samples and dashed blue lines represent the mean (*n* = 2–6 biological replicates). There was a significant effect of genotype on *ca1pase* expression (ANOVA, *P* < 0.001). Significant differences between each OE line and the wild-type are denoted as: •*P* ≤ 0.1; *P ≤ 0.05; ***P ≤ 0.001 (Tukey’s honestly significant difference [HSD] mean-separation test).

Wheat transgenic lines overexpressing the native gene for 2-carboxy-d-arabinitol-1-phosphate phosphatase (CA1Pase) were produced. Based on results from a preliminary experiment with 15 independent lines overexpressing *ca1pase* (first generation, T1) to test for presence of the transgene and enhanced CA1Pase activity, four overexpressing lines (OE1–OE4) were selected for further analysis and grown alongside wild-type plants (Fig. 1A). Based on the presence of the transgene in all the plants investigated, lines OE1 and OE3 were identified as likely homozygous, while lines OE2 and OE4 were verified to be heterozygous (Table 1). For the subsequent analyses, a total of 7 to 10 plants containing the gene of interest were used for each overexpressing line. The five plants that were negative for the presence of the transgene (azygous) were used as an additional negative control and showed a phenotype similar to that of the wild-type plants.

**Table 1.** Qualitative PCR analysis to verify the presence of the transgene for overexpression of *ca1pase* In addition to the experiment described in this manuscript (Experiment 2), a previous experiment was conducted that showed identical results (Experiment 1). Of the 10 plants investigated per line, the transgene was present in all plants in lines OE1 and OE3 (likely homozygous), while it was present in only six to eight plants of lines OE2 and OE4 (heterozygous).

Transgenic Line	No. of Plants Containing the Transgene		Zygosity
	Experiment 1	Experiment 2	
Wild type	0/10	0/10	Negative control
OE1	10/10	10/10	Likely homozygous
OE2	6/10	7/10	Heterozygous
OE3	10/10	10/10	Likely homozygous
OE4	7/10	8/10	Heterozygous

**Figure 2.**
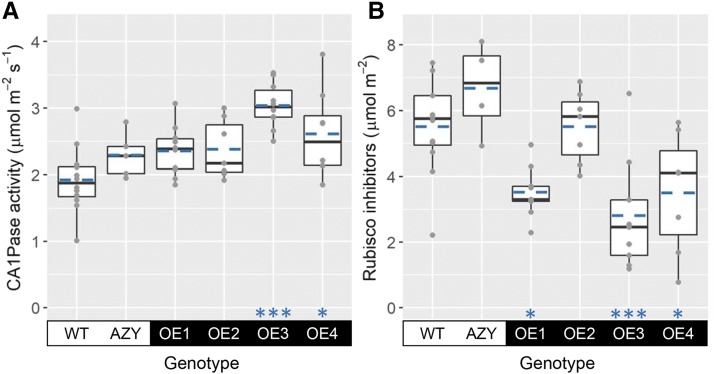
CA1Pase activity and inhibitors of Rubisco activity. CA1Pase activity (A) and quantity of Rubisco tight-binding inhibitors (B) in flag leaves of wheat wild-type plants (WT), negative controls (AZY), and transgenic lines overexpressing *ca1pase* (OE1–OE4). Boxes represent the median and first and third quartiles, whiskers represent the range, symbols represent individual samples, and dashed blue lines represent the mean (*n* = 4–12 biological replicates). There was a significant effect of genotype on CA1Pase activity and Rubisco inhibitors (ANOVA, *P* < 0.001). Significant differences between each overexpressing line and the wild type are denoted as: *P ≤ 0.05; ***P ≤ 0.001 (Tukey’s HSD test).

The expression of *ca1pase* relative to the wild type strongly increased in wheat transgenic lines engineered to overexpress the native gene (OE1–OE4) and was greatest in the OE3 plants (31-fold increase; Fig. 1B). The activity of CA1Pase was greater in both OE3 and OE4 plants compared to the wild type, by 58% and 36%, respectively (Fig. 2A). In OE1 and OE2 plants, while the mean value of CA1Pase activity was higher compared to that observed in wild-type plants, the difference was not statistically significant (Fig. 2A). On the other hand, the quantity of Rubisco tight-binding inhibitors present in the leaves was significantly lower in OE1, OE3, and OE4 than in wild-type plants (with decreases of 35% to 50%), while no significant difference was observed between OE2 and wild-type plants (Fig. 2B).

### Overexpression of *ca1pase* Decreased Rubisco Amount and Activity and Affected Plant Biomass and Grain Yield

**Figure 3.**
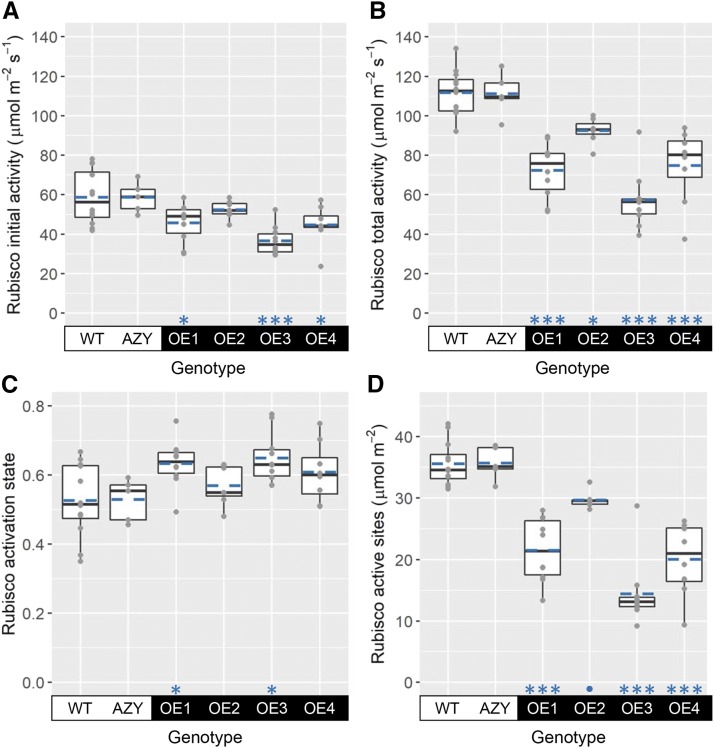
Rubisco activities, activation state, and quantity of active sites. Plots show Rubisco initial (A) and total (B) activities, Rubisco activation state (C), and Rubisco active site content (D) in flag leaves of wheat wild-type plants (WT), negative controls (AZY), and transgenic lines overexpressing *ca1pase* (OE1–OE4). Boxes represent the median and first and third quartiles, whiskers represent the range, symbols represent individual samples, and dashed blue lines represent the mean (*n* = 5–12 biological replicates). There was a significant effect of genotype on Rubisco initial activity (ANOVA, *P* < 0.001), total activity (ANOVA, *P* < 0.001), activation state (ANOVA, *P* < 0.01), and active site content (ANOVA, *P* < 0.001). Significant differences between each OE line and the wild type are denoted as •*P* ≤ 0.1, *P ≤ 0.05, **P ≤ 0.01, and ***P ≤ 0.001 (Tukey’s HSD test).

The activity of Rubisco measured immediately upon extraction of the enzyme from flag leaves (initial activity) and after incubation of the enzyme with CO_2_ and Mg^2+^ to allow for carbamylation of active sites (total activity) was significantly lower in plants overexpressing *ca1pase* compared to wild-type plants (Fig. 3, A and B). The decrease in activity compared to wild-type plants was most marked in the transgenic line with the highest expression of *ca1pase*, OE3 (Fig. 1B). Moreover, total activity decreased to a greater extent than initial activity; Rubisco initial activity in OE3 plants decreased by 38% compared to the wild-type activity, while total activity showed a more marked 49% decrease. Consequently, the activation state of Rubisco, as measured by the ratio of initial to total activities, was 23% higher in OE3 plants compared to wild-type plants (Fig. 3C); a similar increase in Rubisco activation state was observed for the other homozygous line overexpressing *ca1pase*, OE1 (Table 1).

The amount of Rubisco protein (Supplemental Fig. S1A) and, consequently, the number of Rubisco active sites (Fig. 3D) decreased in all lines overexpressing *ca1pase* compared to the wild type, with the greatest decrease occurring in OE3 plants (60% lower than the wild type). These results imply that Rubisco activity (Fig. 3, A and B) was negatively regulated primarily by its reduced amount in plants with higher CA1Pase activity and lower amounts of inhibitors of Rubisco activity (Fig. 2). The decrease in the amount of Rubisco in *ca1pase* overexpressing plants was accompanied by decreases in total soluble protein (up to 25% lower than the wild type; Supplemental Fig. S1B).

**Figure 4.**
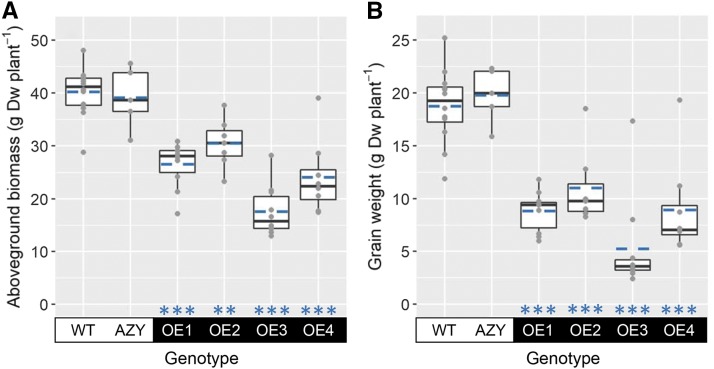
Plant biomass and grain yield. Plots show aboveground biomass (A) and grain weight (B) in wheat wild-type plants (WT), negative controls (AZY), and transgenic lines overexpressing *ca1pase* (OE1–OE4). Boxes represent the median and first and third quartiles, whiskers represent the range, symbols represent individual samples, and dashed blue lines represent the mean (*n* = 5–12 biological replicates). There was a significant effect of genotype on aboveground biomass and grain weight (ANOVA, *P* < 0.001). Significant differences between each overexpressing line and the wild type are denoted as **P ≤ 0.01 and ***P ≤ 0.001 (Tukey’s HSD test).

In addition to the downregulation of Rubisco content and activity in wheat flag leaves in plants overexpressing *ca1pase* (Fig. 3), significant genotypic effects were also observed for total aboveground biomass and grain yield at full maturity (Fig. 4). All the transgenic lines overexpressing *ca1pase* had significantly reduced aboveground biomass and grain yield compared to wild-type plants. OE3 plants showed the greatest decreases in biomass (56% lower than the wild type) and grain yield (72% lower than the wild type). The proportion of biomass allocated to the grain, which is represented by the harvest index, was highly variable (large sd) and not significantly different in the overexpressing lines compared to the wild type (Supplemental Fig. S2A). However, grain produced by plants overexpressing *ca1pase* was lighter than in wild-type plants, as evidenced by the significant decrease in 1,000-grain weight in all overexpressing lines (Supplemental Fig. S2B), with the largest reduction in OE3 (50% lower than the wild type).

In keeping with the observations for OE3 (Figs. 1–4), a correlation analysis across wild-type, azygous, and transgenic plants highlighted significant correlations between *ca1pase* expression, Rubisco biochemistry, and plant productivity (Supplemental Fig. S3). As predicted by our hypothesis, the expression of *ca1pase* in wheat wild-type and transgenic CA1Pase lines was positively correlated with CA1Pase activity and Rubisco activation state and negatively correlated with Rubisco inhibitor content. However, a negative correlation with *ca1pase* expression was also observed for Rubisco active site content, Rubisco initial and total activity, aboveground biomass, and grain yield.

## DISCUSSION

We investigated the impact of increased expression of CA1Pase on the regulation and abundance of Rubisco and on crop yield in wheat. We had expected that reducing the abundance of Rubisco inhibitors (by overexpressing *ca1pase*) would increase the activity of Rubisco and positively impact crop productivity. Our results show the contrary: overexpression of *ca1pase* downregulates Rubisco activity in planta by decreasing the amount of the enzyme, and this negatively affects wheat yield.

The greatest level of *ca1pase* overexpression was observed in transgenic plants of the OE3 line (Fig. 1), which was one of the two lines likely to be homozygous for this trait (Table 1). OE3 plants also showed a highly significant increase in CA1Pase activity and a highly significant decrease in the content of inhibitors of Rubisco activity in the light (Fig. 2). CA1P has been shown to be present in very small amounts in dark-adapted leaves of wheat, especially when compared to CA1P-accumulating leaves of French bean (*Phaseolus vulgaris*; Moore et al., 1991). In contrast, the measured content of alternative inhibitors of Rubisco activity known to occur during the day was equivalent in wheat and French bean (Keys et al., 1995). Given the ability of CA1Pase to dephosphorylate compounds other than CA1P, including diurnal inhibitors of Rubisco activity (Andralojc et al., 2012), it is likely that the lower content of Rubisco inhibitors in illuminated leaves of OE3 plants was a consequence of increased CA1Pase activity dephosphorylating both CA1P and other sugar-phosphate derivatives (Fig. 2; Supplemental Fig. S3).

In agreement with our hypothesis, OE3 plants had lower amounts of Rubisco inhibitors and a higher Rubisco activation state than wild-type plants. However, and contrary to our prediction, the amount and measurable activity of Rubisco was greatly reduced, and grain yield was negatively impacted. In fact, all four *ca1pase* overexpression lines showed significant decreases in Rubisco active sites and total activity in the wheat flag leaf (Fig. 3), as well as significant decreases in aboveground biomass and grain yield (reduced by up to 72% compared to wild-type plants; Fig. 4). Moreover, a strong negative correlation was observed between *ca1pase* expression, content of Rubisco active sites, and grain yield (Supplemental Fig. S3). The increased Rubisco activation state in some of the *ca1pase* overexpression lines partially compensated for the decrease in the content of Rubisco active sites, such that Rubisco initial activity did not significantly correlate with *ca1pase* expression. A negative correlation between Rubisco activation state and content has been reported in multiple studies (see Carmo-Silva et al., 2015 and references therein). For example, this negative correlation was observed in the flag leaves of 64 United Kingdom field-grown wheat cultivars (Carmo-Silva et al., 2017). In that study, Rubisco accounted for >50% of the total soluble leaf protein, and the amount of Rubisco and soluble protein in the leaves decreased as leaves aged, consistent with Rubisco becoming a source of fixed nitrogen for the developing grain (Hirel and Gallais, 2006).

The amount of a given protein in a leaf reflects the balance between its synthesis and degradation (Li et al., 2017). Rubisco is synthesized at fast rates compared to other leaf proteins (Piques et al., 2009). In rice (*Oryza sativa*), Rubisco synthesis has been shown to occur at fast rates, while degradation is minimal until just before the leaf reaches full expansion (Mae et al., 1983; Makino et al., 1984; Suzuki et al., 2001). In wheat plants under normal metabolic conditions, i.e. in the absence of stress and before the onset of senescence, Rubisco is continuously degraded at a slow rate compared to other leaf proteins (Esquível et al., 1998). The degradation of Rubisco in Arabidopsis (*Arabidopsis thaliana*) rosettes has been estimated to occur at a similar rate (0.03–0.08 d^−1^) to that of the total pool of leaf proteins, with a resulting similar protein half-life of ∼3.5 d (Ishihara et al., 2015; Li et al., 2017). A mathematical model developed by Irving and Robinson (2006) suggested that Rubisco degradation is a simple process that follows first-order kinetic principles and is unlikely to be tightly regulated in cereal leaves. On the other hand, translation of both the large and small subunits of Rubisco is tightly coordinated and rapidly adjusted in response to environmental cues (Winter and Feierabend, 1990). This would suggest that the synthesis, rather than degradation, of Rubisco could be impaired in wheat plants overexpressing *ca1pase* (Hirel and Gallais, 2006; Irving and Robinson, 2006).

Evidence suggests that altering the interactions between Rubisco and its molecular chaperone Rca would be a credible strategy to optimize the regulation of Rubisco for enhanced biomass production in the model plant Arabidopsis grown under fluctuating light environments (Carmo-Silva and Salvucci, 2013). In wheat, the response of Rubisco activation to increases in irradiance has been predicted to limit carbon assimilation in fluctuating light environments by up to 21% (Taylor and Long, 2017). These studies indicate that more rapid adjustment of Rubisco activity when a leaf transitions from being shaded to being fully illuminated by sunlight in a canopy could result in significant crop yield increases. Similar to the results reported herein for wheat plants overexpressing *ca1pase*, rice plants overexpressing Rca had a higher Rubisco activation state but lower Rubisco quantity compared to the wild type (Fukayama et al., 2012, 2018). The decreased amounts of Rubisco in rice were not due to changes in the transcription of genes encoding the Rubisco large and small subunits (*rbcL* and RbcS, respectively) or genes encoding chaperones that assist in Rubisco folding and assembly (Rubisco assembly factors 1 [RAF1], RAF2, bundle sheath defective 2 [*BSD2*], and *RbcX*), suggesting that Rubisco amount was modulated by posttranslational factors (Fukayama et al., 2012, 2018). Further research is warranted to examine the hypothesis that the lower amounts of tight-binding phosphorylated compounds in the OE plants may render Rubisco more susceptible to proteolytic breakdown (Khan et al., 1999), thereby enhancing the rate of degradation of the enzyme when plants reach full maturity or experience environmental stress (Suzuki et al., 2001; Ishida et al., 2014).

CA1Pase has been shown to represent a very small proportion of the total leaf protein fraction, even in *P. vulgaris*, a species which has some of the highest amounts of CA1P and of CA1Pase among the plant species studied to date (Moore et al., 1995; Charlet et al., 1997). The same authors showed that measurable CA1Pase activity in wheat is <10% of that observed in *P. vulgaris* (Charlet et al., 1997). The negative effects of *ca1pase* overexpression reported herein suggest that the low abundance of CA1Pase in wheat may have been selected for alongside the relatively large allocation of nitrogen to Rubisco in wheat leaves (Carmo-Silva et al., 2015, 2017; Evans and Clarke, 2019). Significant natural variation in the amount of CA1P and CA1Pase activity has been reported between species and within genera (Vu et al., 1984; Seemann et al., 1985; Moore et al., 1991). Of particular interest in terms of crop improvement is that even among cultivars of soybean (*Glycine max*) and rice, as much as 50% variation has been reported in Rubisco inhibition attributed to CA1P binding (Bowes et al., 1990). This raises the prospect that similar genetic variation in the extent of Rubisco inhibition by phosphorylated compounds may exist in wheat.

That *ca1pase* overexpression diminished the amount of Rubisco active sites in wheat suggests that genetic manipulation of enzymes involved in the regulation of Rubisco may have unexpected consequences, such as downregulation of Rubisco active site content. Further studies to better understand the complexity of Rubisco regulation and genetic variation in the underlying components that affect the activity and content of the carboxylating enzyme will enable a more targeted approach to improve crop yields and resilience to climate change.

## MATERIALS AND METHODS

### Production of CA1Pase Transgenic Lines

Wheat (*Triticum aestivum* ‘Cadenza’) was used for overexpression of 2-carboxy-d-arabinitol-1-phosphate phosphatase (CA1Pase). Plant transformation was carried out by biolistics, as described by Sparks and Jones (2014). To produce the CA1Pase overexpression construct, the full-length *ca1pase* complementary DNA (cDNA) of the wheat D genome was cloned into a vector containing a maize (*Zea mays*) ubiquitin promoter plus intron previously shown to drive strong constitutive expression in wheat (Christensen and Quail, 1996) and nopaline synthase terminator sequences to give pRRes14.ca1pase (Supplemental Fig. S4).

The OE construct was cobombarded with a construct carrying the *bar* selectable marker gene under control of the maize ubiquitin promoter plus intron with a nopaline synthase terminator sequence, pAHC20 (Christensen and Quail, 1996). Transformed calli were selected in tissue culture using phosphinothricin, the active ingredient of glufosinate ammonium-based herbicides. Surviving plants were transferred to soil and grown to maturity. The presence of the transgene was confirmed by PCR using primers as described in Supplemental Table S1. The transformation process generated 15 overexpressing lines; resulting T1 plants of each transgenic line were allowed to self-pollinate to produce the T2 generation, which was used in this study. Transformed plants were selected by screening for gene presence and expression using qualitative PCR analysis (Supplemental Table S1). Four independent T2 lines (OE1–OE4) were selected based on enhanced CA1Pase activity in earlier experiments with T1 and T2 plants.

### Plant Growth Conditions

Plants were grown in semicontrolled conditions in a glasshouse at the Lancaster Environment Centre with minimum temperatures set to 24°C day/18°C night. The observed maximum daily temperatures were typically higher than 24°C and occasionally exceeded 30°C on very sunny days. The photoperiod was set to 16 h with supplemental lighting provided when external light levels fell below 200 µmol m^−2^ s^−1^. Seeds were sown on June 27, 2017 into 3 L round pots with a 3:1 mixture of special wheat mix growth media (Petersfield compost, Hewitt & Son) and silver sand (Kelkay Horticultural Silver Sand, Royal Horticultural Society). Initial experiments tested the pot size and medium composition, enabling optimization of the growth conditions. Plants, including 12 of the wild type and 10 of each transgenic line (OE1–OE4), were distributed according to a split-plot design with equal replicates per genotype. All pots were kept well watered throughout the experiment.

Leaf samples for genotyping were taken from 3-week-old plants. Samples for biochemical analyses were taken from the flag leaf of the main tiller of each plant prior to complete ear emergence (growth stage Zadoks 4.5–5.5; Zadoks et al., 1974), collected 4–5 h after the beginning of the photoperiod and rapidly snap-frozen in liquid nitrogen followed by storage at −80°C until analysis.

### Genotyping to Evaluate the Presence or Absence of DNA of Interest

Leaf samples were taken from 3-week-old plants, placed directly into wells of a deep 96-well plate (Life Technologies) and freeze-dried for 2 d. Leaf material was ground using a Tissue Lyser (Retsch MM200, Qiagen) with two 5 mm ball bearings per well. DNA was extracted following the protocol described by Van Deynze and Stoffel (2006). PCR was completed in 20 µL reactions (per the manufacturer’s instructions; GoTaq DNA Polymerase, Promega). Primers and PCR conditions are listed in Supplemental Table S1. Positive controls using the plasmid were included. PCR fragments were separated in 0.8% (w/v) agarose gels and visualized in the presence of SYBR safe DNA gel stain (Invitrogen, Thermo Fisher Scientific). This enabled verification of homozygous lines (OE1 and OE3) and identification of positive versus negative plants for presence of the transgene in the heterozygous lines (OE2 and OE4). The five plants that showed no evidence of presence of the transgene (azygous) were subsequently used as negative controls alongside the wild type.

### Reverse-Transcription Quantitative PCR

To evaluate the expression of *ca1pase*, mRNA was extracted using a NucleoSpin Tri Prep kit (Macherey-Nagel) including DNase treatment. RNA concentration and quality were determined via a spectrometer (SpectraStar Nano, BMG Labtech). A subsample of 1 µg RNA was used for cDNA synthesis using the Precision nanoScript 2 Reverse Transcription kit (Primer Design) according to the manufacturer’s instructions. Reverse-transcription quantitative PCR (qPCR) was performed with the PrecisionPLUS qPCR Master Mix kit (Primer Design) containing cDNA (1:5 dilution) and the primer pair (Supplemental Table S1) in a Mx3005P qPCR system (Stratagene, Agilent Technologies). Melting curves were also completed. Primer efficiency was analyzed based on a cDNA dilution series with mean primer efficiency estimated using the linear phase of all individual reaction amplification curves and calculated according to Pfaffl (2001). The succinate dehydrogenase (UniGene Cluster ID Ta.2218) and ADP-ribosylation factor (Ta.2291) genes were used as reference genes to normalize gene expression (Paolacci et al., 2009; Evens et al., 2017). The normalized relative quantity (NRQ) of expression was calculated in relation to the cycle threshold (CT) values and the primer efficiency (E) of the target gene (X) and the reference genes (N), based on Rieu and Powers (2009): NRQ = (EX)^−CT,X^/(EN)^−CT,N^.

### Protein Extraction and Enzyme Activity Assays

Total soluble protein was extracted according to Carmo-Silva et al. (2017) with slight modifications. Flag leaf samples were ground in an ice-cold mortar and pestle in the presence of extraction buffer (50 mm Bicine-NaOH, pH 8.2, 20 mm MgCl_2_, 1 mm EDTA, 2 mm benzamidine, 5 mm ε-aminocaproic acid, 50 mm 2-mercaptoethanol, 10 mm dithiothreitol, 1% [v/v] protease inhibitor cocktail [Sigma-Aldrich], 1 mm phenylmethylsulphonyl fluoride, and 5% [w/v] polyvinylpolypyrrolidone). The homogenate was clarified by centrifugation at 14,000*g* for 1 min at 4°C. The supernatant was used to measure Rubisco activities and amount, CA1Pase activity, and total soluble protein concentration (Bradford, 1976).

Rubisco activities were determined immediately upon extraction via incorporation of ^14^CO_2_ into stable sugars as described by Carmo-Silva et al. (2017). The initial activity was initiated by adding supernatant to the reaction mixture: 100 mm Bicine-NaOH, pH 8.2, 20 mm MgCl_2_, 10 mm NaH^14^CO_3_ (9.25 kilobecquerel μmol^−1^), 2 mm KH_2_PO_4_, and 0.6 mm RuBP. For the total activity, extract was incubated with the assay buffer (without RuBP) for 3 min prior to assaying, and the reaction was started by addition of 0.6 mm RuBP to the mixture. Reactions were performed at 30°C and quenched after 30 s by addition of 100 μL of 10 m formic acid. To quantify the acid-stable ^14^C, assay mixtures were dried at 100°C, and the residue was redissolved in deionized water and mixed with scintillation cocktail (Gold Star Quanta, Meridian Biotechnologies) prior to liquid scintillation counting (Packard Tri-Carb, PerkinElmer). All assays were conducted with two analytical replicates. Rubisco activation state was calculated from the ratio (initial activity/total activity) × 100. The amount of Rubisco was quantified in the same supernatant by a [^14^C]CA 1,5-bisphosphate binding assay (Whitney et al., 1999).

CA1Pase activity was measured by the formation of Pi following the method described by Van Veldhoven and Mannaerts (1987), with modifications as in Andralojc et al. (2012). The assay was initiated by adding supernatant to the reaction mixture: 50 mm Bis-tris propane, pH 7.0, 200 mm KCl, 1 mm EDTA, 1 mm ε-aminocaproic acid, 1 mm benzamidine, 10 mm CaCl_2_, 0.5 mg/mL bovine serum albumin, 1% (v/v) protease inhibitor cocktail (Sigma-Aldrich), and 0.5 mm CRBP. A negative control without CRBP was included. After 60 min, the activity assay was quenched with 1 m trichloroacetic acid; the mixture was centrifuged at 14,000*g* for 3 min to sediment protein residues and the supernatant was mixed with 0.44% (w/v) ammonium molybdate in 1.6 m H_2_SO_4_ and, after 10 min, 0.035% (w/v) malachite green in 0.35% (w/v) poly(vinyl) alcohol. After 60 min at room temperature, the absorbance at 610 nm was determined and the quantity of Pi calculated based on a standard curve with K_2_HPi.

### Quantification of Rubisco Inhibitors

Tight-binding inhibitors of Rubisco activity were quantified as described by Carmo-Silva et al. (2010). Leaf samples were ground to a fine powder in liquid nitrogen and inhibitors extracted following further grinding with 0.45 m trifluoroacetic acid. After thawing and centrifugation (14,000*g* for 5 min at 4°C), a subsample of the supernatant (20 µL) was incubated for 5 min with 10 µg of activated wheat Rubisco (previously purified as described by Orr and Carmo-Silva, 2018) in 100 mm Bicine-NaOH, pH 8.2, 20 mm MgCl_2_, and 10 mm NaH^12^CO_3_. The extent of Rubisco activity inhibition was measured in the presence of complete assay buffer with 100 mm Bicine-NaOH, pH 8.2, 20 mm MgCl_2_, 10 mm NaH^14^CO_3_ (18.5 kilobecquerel µmol^−1^), and 0.4 mm RuBP. The inhibitor content was determined by reference to a standard curve with known quantities of CA1P in trifluoroacetic acid, which had been incubated with activated Rubisco exactly as described above and prepared alongside the sample reactions.

### Biomass and Yield Traits

Plant aboveground biomass was determined at full physiological maturity (growth stage Zadoks 9.1–9.2; Zadoks et al., 1974). Tillers and spikes were counted, and vegetative biomass (leaves and stems) was dried at 65°C until constant weight was attained. Ears were threshed (Haldrup LT-15, Haldrup GmbH), and a seed subsample of ∼3 g was used to determine water content and to estimate the number of seeds using the phone app SeedCounter (Komyshev et al., 2017) to calculate the 1,000-grain weight. The harvest index was estimated by the ratio between the dry weights of grain and aboveground biomass per plant.

### Statistical Analysis

One-way ANOVA was used to test statistical significance of differences between means of each trait for the six genotypes. Where a significant genotype effect was observed, a Tukey posthoc test was used for multiple pairwise comparisons. Statistical analyses were performed in R (version 3.3.3; R Core Team, 2016) and RStudio (version 1.0.153; RStudio Team, 2015). Box and whiskers plots were prepared using ggplot2 (Wickham, 2016); boxes show medians and first and third quartiles (25th and 75th percentiles), and whiskers extend from the hinge to the largest or smallest value, no further than 1.5 times the interquartile range (distance between the first and third quartiles). Symbols represent individual data points and dashed lines represent the mean values.

### Accession Numbers

Sequence data for CA1Pase can be found in the GenBank data library under accession number HE603918 (Phytozome gene reference Traes_4DS_1860220B9).

### Supplemental Data

The following supplemental materials are available.

**Supplemental Figure S1.** Rubisco and total soluble protein content.
**Supplemental Figure S2.** Harvest index and 1,000-grain weight.
**Supplemental Figure S3.** Correlation matrix showing the significance of pairwise linear correlations between *ca1pase* expression, Rubisco biochemistry and plant productivity traits.
**Supplemental Figure S4.** Construct used for wheat plant transformation to overexpress *ca1pase*.
**Supplemental Table S1.** Primers and PCR conditions for DNA and gene expression analysis.
